# Systemic Maternal Human sFLT1 Overexpression Leads to an Impaired Foetal Brain Development of Growth-Restricted Foetuses upon Experimental Preeclampsia

**DOI:** 10.1155/2022/3024032

**Published:** 2022-06-02

**Authors:** Rebekka Vogtmann, Lilo Valerie Burk, Meray Serdar, Rainer Kimmig, Ivo Bendix, Alexandra Gellhaus

**Affiliations:** ^1^Department of Gynaecology and Obstetrics, University Hospital Essen, 45147 Essen, Germany; ^2^Department of Paediatrics I, Neonatology & Experimental Perinatal Neurosciences, University Hospital Essen, 45147 Essen, Germany; ^3^Centre for Translational Neuro- and Behavioural Sciences, C-TNBS, Faculty of Medicine, University Duisburg-Essen, 45147 Essen, Germany

## Abstract

The pregnancy disorder preeclampsia (PE) is characterized by maternal hypertension, increased level of circulating antiangiogenic soluble fms-like tyrosine kinase-1 (sFLT1), and reduced placental perfusion, leading to foetal growth restriction (FGR) and preterm birth. All these adverse effects are associated with neurocognitive disorders in the offspring. However, the direct interplay between increased antiangiogenesis during PE and disturbed foetal brain development independent of prematurity has not been investigated yet. To examine foetal brain development in sFLT1-related PE, hsFLT1/rtTA-transgenic mice with systemic (maternal or maternal/fetoplacental) human sFLT1 (hsFLT1) overexpression since 10.5 days postconception (dpc) were used, and histological and molecular analyses of foetal brains were performed at 18.5 dpc. Consequences of elevated hsFLT1 on placental/foetal vascularization and hypoxia of placentas and foetal brains were analysed using the hypoxia markers pimonidazole and hemeoxygenase-1 (HO-1). Immunohistochemical analysis revealed increased hypoxia in placentas of PE-affected pregnancies. Moreover, an increase in HO-1 expression was observed upon elevated hsFLT1 in placentas and foetal brains. PE foetuses revealed asymmetrical FGR by increased brain/liver weight ratio. The brain volume was reduced combined with a reduction in the cortical/hippocampal area and an increase of the caudate putamen and its neuroepithelium, which was associated with a reduced cell density in the cortex and increased cell density in the caudate putamen upon hsFLT1 overexpression. Mild influences were observed on brain vasculature shown by free iron deposits and mRNA changes in Vegf signalling. Of note, both types of systemic hsFLT1 overexpression (indirect: maternal or direct: maternal/fetoplacental) revealed similar changes with increasing severity of impaired foetal brain development. Overall, circulating hsFLT1 in PE pregnancies impaired uteroplacental perfusion leading to disturbed foetal oxygenation and brain injury. This might be associated with a disturbed cell migration from the caudate putamen neuroepithelium to the cortex which could be due to disturbed cerebrovascular adaption.

## 1. Introduction

The hypertensive placenta-specific pregnancy disorder preeclampsia (PE) increases the risk of perinatal morbidity and mortality in mothers and their children and is highly associated with long-term health issues in both. The placental dysfunction during PE is characterized by disturbed oxygenation due to an impaired uteroplacental vascularisation, which is often combined with foetal growth restriction (FGR) and/or preterm birth [1–3]. Pathological findings of PE-specific marker systems include altered placental expression of angiogenic factors such as the antiangiogenic biomarker soluble fms-like tyrosine kinase-1 (sFLT1) and its accumulation in maternal serum [4]. Since elevated sFLT1 levels are present in the serum of pregnant women before symptoms appear, this factor is already used as a clinical biomarker to predict PE [5, 6]. So far, the only effective therapy is the removal of the placenta and thereby an induction of a preterm birth combined with all adverse consequences for the postnatal offspring's development [7].

Recent studies showed that higher levels of sFLT1 in early-onset PE significantly correlate with an abnormal mean pulsatility index of uterine arteries, supporting the hypothesis that high sFLT1-levels are associated with disturbed uteroplacental vascularisation in PE [8–10]. Our own previous work using an experimental transgenic human sFLT1- (hsFLT1-) related PE/FGR mouse model clearly showed that elevated hsFLT1 levels in maternal circulation resulted in placental dysfunction characterized by increased placental hypoxia, shown by an increase in hypoxia-inducible factors (*Hif1α*, *Hif2α*), leading to foetal growth restriction already starting at the end of the second trimester during pregnancy with viable and nonviable foetuses. Our previous results supported the hypothesis that this was due to reduced uteroplacental vascularization including impaired spiral arterial remodelling and foetal vessel development in the placental labyrinth [11, 12].

According to the Barker hypothesis, the foetus can be preprogrammed for conditions that will occur later in life, named “foetal programming” [13]. Additionally, retrospective human studies have shown that children born with PE and/or FGR have a higher risk to develop not only temporary and continuing metabolic or cardiovascular diseases but also neurological impairments and cognitive disorders in later life [1, 2, 14–16].

Moreover, the role of the placenta as a master regulator in protecting and shaping the foetal brain becomes of increased importance and is named as a new research topic of “neuroplacentology” [14]. Placental dysfunction or pathology has been linked to impaired neurodevelopment, but the underlying mechanisms remain largely unknown, although the causal relationship between PE/FGR and adverse neurodevelopmental outcome is still a matter of debate [17–19]. Physiological brain development is highly dependent on adequate vascularisation not only for oxygen and nutrient supply but also for creating the scaffold necessary for neuronal and oligodendroglial migration [20, 21]. The brain is a complex organ, not only with different anatomical regions with their own specific functions but also with unique developmental trajectories that begin during foetal development proceeding until the early postnatal age [22]. Thereby, the different developmental stages overlap and include diverse processes of cell proliferation, division, differentiation, migration, and connectivity, which are precisely controlled in a spatiotemporal manner. Thus, any disturbance of these processes during pregnancy, for example, due to undernutrition or hypoxia, might affect foetal brain function [23].

Even though elevated systemic sFLT1 levels have been detected in a prospective study in young adult individuals born preterm from hypertensive pregnancies, it remains unclear whether these can be directly attributed to high sFLT1 levels in their mothers [24]. Though recently suggested by Lara and colleagues [25] that alterations in sFLT1 levels in preeclamptic pregnancies will affect foetal cerebrovascular function and neurodevelopment, direct effects of elevated antiangiogenic molecules like sFLT1 on brain development and vascularisation/oxygenation are still missing.

Taking into account that human sFLT1 (hsFLT1) overexpression revealed a negative impact on fetoplacental [11] and uteroplacental vascularization [12] as well as foetal body weight gain during pregnancy [11, 12] in our hsFLT1-transgenic PE mouse model, we conjectured that systemic hsFLT1 overexpression impairs foetal neurodevelopment during pregnancy due to undernutrition and underperfusion of the placenta and foetus and thereby might promote long-lasting neurodevelopmental deficits in PE offspring independent of prematurity.

## 2. Materials and Methods

### 2.1. Ethics Statement

As already done and stated in [11, 12], all animal experiments were approved and performed according to the local ethics committee and the German laws for animal protection (State Agency for Nature, Environment and Consumer Protection, North Rhine-Westphalia (LANUV), G1265/12 and G1644/17).

### 2.2. Animals

The mouse line generation, underlying genetics of hsFLT1/rtTA mice and housing of the mice, is previously detailed described in [11, 12]. Of note, the mating strategy in the hsFLT1/rtTA model in the current study was slightly adjusted to obtain more single-transgenic foetuses in the experimental set-up. For the PE and control (Ctrl) group, hsFLT1/rtTA females (homozygous for hsFLT1 and heterozygous for rtTA) were mated with single-transgenic hsFLT1 males (homozygous for hsFLT1 and wild type (wt) for rtTA), and for the doxycycline (Dox) Ctrl group, single-transgenic hsFLT1 males and females were mated. Mating was done over one night, and the next day was set as 0.5 day postconception (dpc). Treatment and thus hsFLT1 overexpression were induced from midpregnancy (10.5 dpc) until nearly the end of the pregnancy (18.5 dpc). Therefore, dams received either 2 mg/ml Dox solved in 3% sucrose water in the PE and Dox Ctrl group or sucrose water only in the Ctrl group. The PE group was further subdivided by the foetal rtTA genotype, in which double transgenic hsFLT1/rtTA foetuses/placentas (PE het: homozygous for hsFLT1 and heterozygous for rtTA) can also express hsFLT1 themselves, whereas single transgenic foetuses/placentas (PE wt: lacking the rtTA allele) cannot. According to this, different variations of hsFLT1 expression were achieved in the PE group according to the foetal rtTA genotype.  Table 1 displays the differences between the maternal and foetal experimental groups and the origin of hsFLT1 expression in PE wt (exclusive maternal) compared to the PE het group (maternal and fetoplacental). Sampling of foetuses and foetal brains was done at 18.5 dpc (Ctrl: *n* = 9, Dox Ctrl: *n* = 4, PE: *n* = 11 individual pregnancies; Ctrl: *n* = 42, Dox Ctrl: n =19, PE wt: *n* = 35, PE het: *n* = 19 individual foetuses/foetal brains).

### 2.3. Tissue Preparation

Tissue preparation was done as previously described [11, 12]. At 18.5 dpc, pregnant mice were sacrificed. Maternal blood was taken to measure hsFLT1 serum levels. Foetuses and foetal brains and livers were isolated and weighed, to calculate the brain to liver ratio. Organs were either frozen and stored at −80°C (for RNA, DNA) or immediately fixed in ice-cold 4% (*w*/*v*) paraformaldehyde for 24 h at 4°C and stored in 70% (*v*/*v*) ethanol at 4°C until being embedded in paraffin standard procedures (for morphology).

### 2.4. Morphological Analysis of Hypoxia with Pimonidazole Hydrochloride

For morphological analysis of hypoxia in the murine tissues, pregnant mice were injected with 0.1 ml of 0.06 mg/g Pimonidazole hydrochloride (Hypoxyprobe, Inc., Burlington, USA) in 0.9% NaCl intraperitoneally 30 minutes prior to anaesthesia with isoflurane and execution at 18.5 dpc. Tissues were processed, embedded, and cut as described above. DAB staining was done according to the manufacturer's protocols with “Vectastain Elite ABC-HRP KIT PK-6100” (Vector Laboratories) and “Peroxidase Substrate KIT DAB” (Dako). Three to four slides per placenta and foetal brain with four serial sections each were used. Briefly, incubation with the primary antibody anti-pimonidazole mouse IgG1 monoclonal MAb1 (placentas: 1 : 100 in 0.5% PBS/BSA; foetal brains: 1 : 50) was done overnight at 4°C. The biotinylated polyclonal goat anti-mouse IgG was applied (1 : 100 in 0.5% PBS/BSA) for one hour at room temperature. Slides were incubated for 30 minutes with Vectastain Elite® ABC-Kit (Vector Laboratories, Burlingame, USA) for signal amplification, and signal detection was done by using the liquid DAB+ substrate chromogen system (# K3468; Dako, Carpinteria, CA, USA).

Stained images were converted into TIFF files with ImageScope (Leica Biosystems, Wetzlar, Germany) for further analysis using Fiji/ImageJ [26]. Images were converted into binary images, and a black and white threshold was set at 200/255 for placental and 230/250 for foetal brain sections. The staining intensity of the whole placenta/brain or only placental parts (labyrinth, decidua, and spongiotrophoblast) were calculated. The mean of five sections was determined, and graphical analysis was performed using GraphPad Prism 9.2.0 (La Jolla, USA).

### 2.5. Serum hsFLT1 Measurements via Brahms Kryptor

Serum was prepared from whole blood samples, and hsFLT1 concentrations were measured with a BRAHMS KRYPTOR compact PLUS analyser as previously described [11, 12].

### 2.6. Image Analysis

Histology was assessed on formalin-fixed and paraffin-embedded samples. Foetal brains were sectioned coronally at 5 *μ*m and mounted on Superfrost Plus Slides (R. Langenbrinck, Emmendingen, Germany). Scanning at 20x or 40x magnification was done by the Westdeutsche Biobank, University Hospital Essen, Germany, and image processing was done as previously described [11, 12]. For histological assessment, the whole foetal brain volume (mm^3^) and brain compartment sizes (mm^2^) were measured in two serial sections at seven different parts with 100 *μ*m intervals between +2.20 mm and +2.90 mm rostral, according to the mouse developing atlas [27], and integrated to obtain the volume (HE), or three to four serial sections in two brain regions were analysed (nucleus count in HE andiron deposits in Perls). For quantitative image analysis of special stains (HE and Perls), the original exported Red-Green-Blue (RGB) image was split into an 8-bit image containing exclusively the information of one colour of each staining: haematoxylin (blue), Perls Prussian Blue (blue), or Periodic Acid Schiff (magenta). Afterwards, each image was thresholded to get a binary black and white image, showing in white the staining of interest and in black the background. By using the “watershed” algorithm, single nuclei (recognized by haematoxylin) or iron particles (recognized by Perls) were separated, and the number and size were calculated with the “analyse particle” command.

### 2.7. Haematoxylin and Eosin Staining

To assess the general brain morphology, haematoxylin and eosin (HE) stain was used as described in [11] for placental analyses. In short, sections were deparaffinized, rehydrated, 5 min incubated with haematoxylin solution (Thermo-Scientific, Pittsburgh, USA), and shortly washed in tap water and ethanol (70%)/HCl (0.5%) solution for differentiation, followed by 5 min bluing in flowing tap water. Afterwards, sections were incubated for 3 min in eosin solution (#7089.1, Carl Roth, Karlsruhe, Germany) and washed several times in 96% ethanol for differentiation, followed by a standard dehydration procedure and mounting in xylene mountant. With HE stain, nuclei are displayed in blue and cytoplasm is displayed in red. HE stain was used for morphometric analyses of brain volume and brain region areas (about 14 images per sample) as well as nucleus counting (nine images per sample).

### 2.8. Perls Prussian Blue Reaction

Perls Prussian Blue reaction was used to detect and identify ferric (Fe^3+^) as a sign for haemorrhage. Therefore, Perls' Prussian Blue (Perls) Iron Special Stain Kit (38016SS7, Leica Biosystems) was used according to the manufacturer's protocol. Briefly, 4% ferrocyanide solution and 4% acidified water were mixed immediately before incubating slides for 45 min then counterstained with 1% neutral red. Number and size of Perls-positive particles were calculated in different brain regions (nine images each sample).

### 2.9. Genomic DNA Isolation, Genotyping, and Sex Determination

Genomic DNA isolation, genotyping, and sex determination were done as previously described [11, 12], with the appropriate primers listed in Table 2.

### 2.10. RNA Extraction, cDNA Synthesis, and Quantitative Polymerase Chain Reaction

RNA extraction, cDNA synthesis, and quantitative polymerase chain reaction (qPCR) were performed as previously described [11, 12]. In short, total RNA was extracted from one hemisphere (excluding the cerebellum) with the RNeasy Mini Plus Kit (#74134, Qiagen, Hilden, Germany) according to the manufacturer's protocol. The quality and quantity of RNA were verified with *μ*Cuvette G1.0 and BioPhotometer Plus (Eppendorf, Hamburg, Germany). Only RNA with 260/280 = ~2.0 and 260/230 = 2.0 − 2.2 was used for complementary DNA (cDNA) synthesis. cDNA was synthesized from 2 *μ*g RNA. Gene expression was measured from 1 *μ*l cDNA with 19 *μ*l of the PowerUP SYBR Green Master Mix (#A25742; Applied Biosystems, Foster City, CA, USA) and the ABI Prism 7300 Sequence Detection System (Applied Biosystems) with a standard PCR program. The quantitative PCR (qPCR) analyses were carried out in triplicate. The amount of cDNA in each sample was normalized to glyceraldehyde-3-phosphate dehydrogenase (Gapdh) and *β*-Actin (Actb) as housekeeping genes and final gene expression analysis was done by the ∆∆CT method (for angiogenesis markers, brain cell markers and cell adhesion markers). The amount of cDNA in each sample was normalized to *β*-Actin (Actb) as a housekeeping gene and final gene expression analysis was done by standard curve method (for HO-1).

Primer design was done as previously described [11, 12] with Primer3, and primer sequences are listed in Table 2. The primers for *β*-Actin, Gapdh, hsFLT1, (s)Flt-1, Flk-1, Flt-4, Plgf, Vegfa-d, HO-1, and Cd31 for qPCR analyses and hsFLT1, Col1a1, rtTA, IL-3, and Sry were previously generated used by us in [11, 12]. All other used primers were newly generated for this study.

### 2.11. Immunoblot Analysis

Proteins were isolated from placental and foetal brain tissues, and immunoblot analysis was done as previously described [12]. For normalization, each blot of one type of tissue was run with the same sample of reference. Nitrocellulose membranes were incubated with an antibody specific for HO-1 (1 : 1500; #NBP1-31341, Novus biologicals) in 2% (*w*/*v*) milk powder solved in TBS-T at 4°C overnight. Membranes were incubated with 1 : 5000 diluted goat anti-rabbit IgG-HRP (p0448, DAKO), in 0.5% (*w*/*v*) milk powder in TBST at room temperature. *β*-Actin was used as a reference gene for normalization of each blot (*β*-Actin peroxidase) (1 : 200.000; A3854, Sigma).

### 2.12. Statistics

Statistical analysis was done as previously described [11, 12]. Since no normal distribution was found for all experimental groups, Kruskal–Wallis and Dunn's multiple comparison tests were used to compare four experimental groups and Mann–Whitney to compare two groups (*p* value of 0.05 or less was indicated with ∗, ∗∗*p* < 0.01, and ∗∗∗*p* < 0.001). Data is presented as box plot, with median and interquartile range ± minimum to maximum. Sample size (*n*) is listed under each graph, respectively. Statistics was done with GraphPad Prism software version 5.01 (GraphPad, La Jolla, CA, USA).

## 3. Results

### 3.1. Systemic Human sFLT1 Overexpression Induces Foetal Growth Restriction in Mice

**Figure 1:**
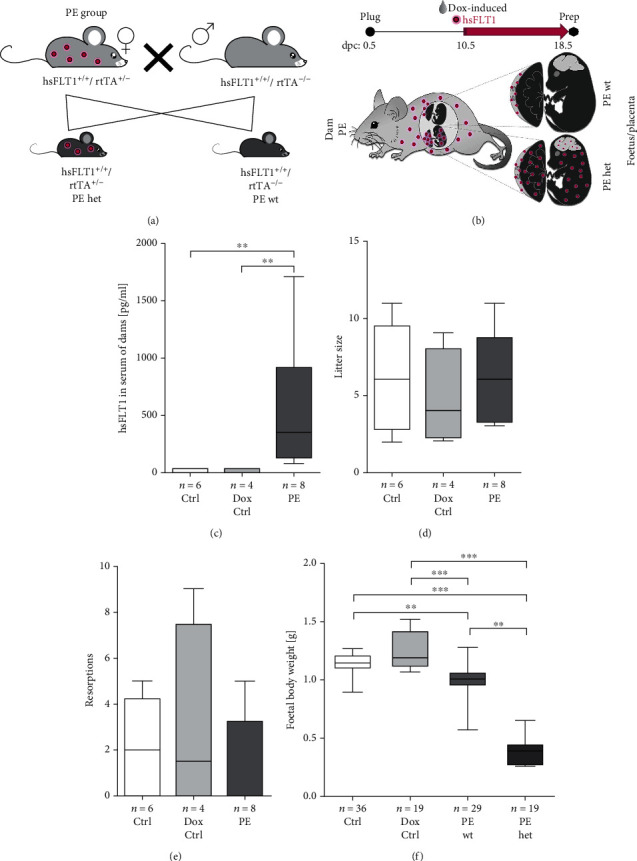
Systemic human sFLT1 (soluble fms-like tyrosine kinase-1) overexpression results in reduced foetal body weight at the end of the pregnancy. (a) In the experimental preeclampsia (PE) group, hsFLT1 (human sFLT1)/rtTA (reverse tetracycline-controlled transactivator) females (hsFLT1+/+ and rtTA+/−) were mated with hsFLT1-transgenic males, lacking the rtTA allele (hsFLT1+/+ and rtTA−/−). In one PE pregnancy, either foetuses developed with the genotype hsFLT1+/+/rtTA+/− (PE het; heterozygous for rtTA) or foetuses with the genotype hsFLT1+/+/rtTA−/− (PE wt; wild type for rtTA). Two control (Ctrl) groups were performed, first with the same maternal genotype (hsFLT1+/+ and rtTA+/−) and the same mating strategy as for the PE group, named Ctrl group, and second by mating single-transgenic hsFLT1 males and females lacking the rtTA allele (hsFLT1+/+ and rtTA−/−), which can be used to test for doxycycline (Dox) side effects, named Dox Ctrl group. (b) At 10.5 until 18.5 days postconception (dpc), dams were treated either with doxycycline (Dox) and sucrose (PE/Dox Ctrl) or with sucrose only (Ctrl). Consequently, in PE dams, systemic hsFLT1 overexpression was induced from midgestation (10.5 dpc) until the end of the experiment (18.5 dpc). Since Dox passes the placental barrier, for foetal analyses, the maternal PE group was further subdivided into PE het foetuses, which developed under combined systemic maternal and fetoplacental hsFLT1 overexpression, and PE wt foetuses, which developed under exclusive maternal hsFLT1 overexpression. The difference in the origin of hsFLT1 overexpression between PE wt (maternal) and PE het (maternal and fetoplacental) is illustrated with magenta dots. (c) hsFLT1 was exclusively present in serum of induced dams (PE). (d) There were no differences in the litter size or (e) resorption rate between the experimental groups. (f) At 18.5 dpc, the foetal body weight was reduced in both types of hsFLT1 overexpression (exclusive maternal (PE wt) and combined maternal and fetoplacental (PE het)) compared to both controls. Data are presented as box plot with median and interquartile range ± upper/lower extreme; sample size *n* of individual tested dams, litters, or foetuses is listed under each graph, respectively; Kruskal-Wallis combined with Dunn multiple comparisons test; ^∗∗^*p* < 0.01 and ^∗∗∗^*p* < 0.001.

As previously already established and characterized by us [11, 12], in transgenic hsFLT1/rtTA mice, hsFLT1 was systemically induced in midpregnancy (10.5 dpc) in dams and foetuses (PE het) or in dams only (PE wt) depending on the foetal genotype. Of note, within the same pregnancy, PE dams carried both types of foetuses in parallel, foetuses either having both transgenic alleles hsFLT1 and rtTA and express hsFLT1 also by themselves (PE het) or foetuses lacking the rtTA allele and did not express hsFLT1 (PE wt). Accordingly, for foetal analyses, a discrimination between the effects of exclusive maternal hsFLT1 overexpression and combined maternal and fetoplacental hsFLT1 was possible (foetus: PE subdivided into PE wt and PE het), whereas maternal hsFLT1 levels, litter sizes, or resorption rates could only be characterized for the whole pregnancy (group named PE without subgroups) (Table 1). The mating strategy, experimental set-up, and the difference in the origin of hsFLT1 overexpression between PE wt and PE het group are illustrated in Figures 1(a) and 1(b). At the end of the pregnancy, median maternal hsFLT1 serum levels were 351.7 pg/ml in PE dams. No induction or leakiness was detectable in both uninduced controls (Ctrl and Dox Ctrl) (Figure 1(c)). No significant differences were seen in the litter size, as well in the total number of observed resorptions (Figures 1(d) and 1(e)). At 18.5 dpc, foetuses of the PE group showed different stages of growth restriction dependent from different origins of hsFLT1 overexpression compared to both control groups (Ctrl and Dox Ctrl). Thereby, the foetal body weight was reduced in both types of hsFLT1 overexpression in the PE subgroups (Figure 1(f)). Upon exclusive maternal hsFLT1 overexpression (PE wt), foetuses revealed a mild reduction in body weight but still leading to viable foetuses. In contrast, combined maternal and fetoplacental hsFLT1 overexpression (PE het) led to a strong reduction in foetal body weight and nonviability of the foetuses at the end of the pregnancy.

Thus, experimental PE induced via systemic hsFLT1 resulted in different severity levels of growth restriction during pregnancy, showing viable and nonviable foetuses, depending on the origin of the hsFLT1 overexpression.

### 3.2. Systemic Human sFLT1 Overexpression Disturbs Uteroplacental Vascularisation Resulting in Oxygen Deprivation and Increased Oxidative Stress-Responsive HO-1 Expression

**Figure 2:**
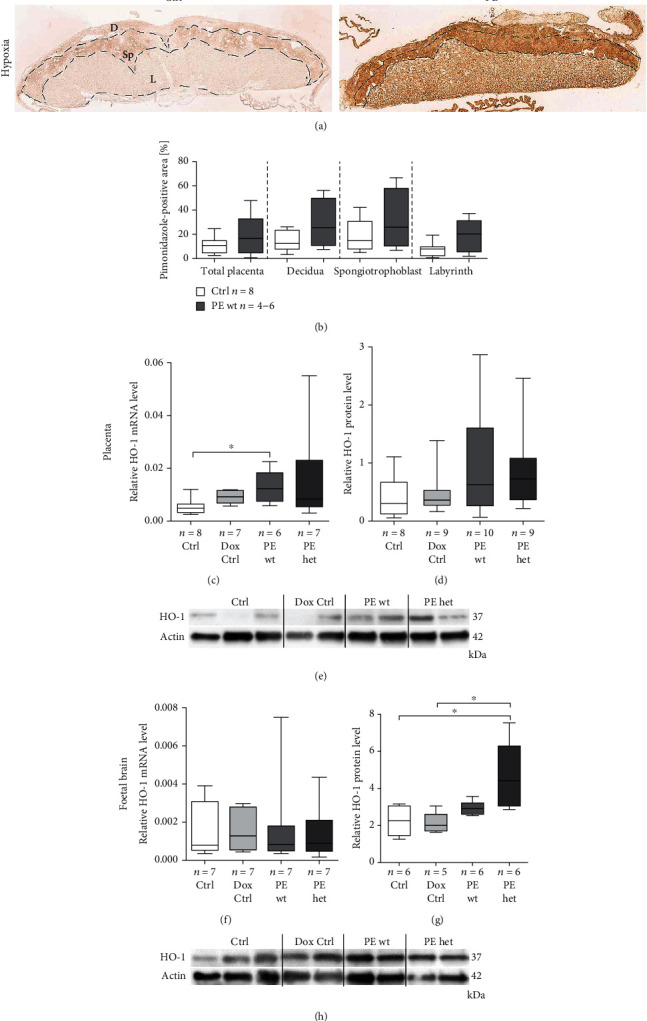
Morphological analysis of increased hypoxia in placentas and foetal brains upon systemic human sFLT1 (soluble fms-like tyrosine kinase-1) overexpression at the end of the pregnancy. (a) Overview of anti-pimonidazole-stained placentas at 18.5 dpc; control group (Ctrl) and preeclampsia group (PE wt); scale bar: 900 *μ*m. (b) Quantification of pimonidazole-positive area (%) revealed increased hypoxia in total PE placental area (PE wt) and in all separate compartments compared to control placentas (Ctrl). Data are presented as box plot with median, interquartile range, and min/max; sample size *n* is listed for each group; Mann-Whitney test was performed. D: decidua; L: labyrinth; Sp: spongiotrophoblast. (c–h) HO-1 (hemeoxygenase-1) gene and protein expression in placentas and foetal brain tissue upon hsFLT1 overexpression. (c) At 18.5 dpc, *HO-1* mRNA expression (normalized to *Actin* mRNA) in PE wt and het placentas was significantly increased, whereas its expression in foetal brains is not affected (f). HO-1 protein level (ca. 37 kDa) normalized to Actin protein level (42 kDa) was increased by trend in placental tissues (d, e) and significantly increased in foetal brains (g, h) in both PE groups (PE wt and het). Data are presented as median, interquartile range, and min/max. Sample size *n* is listed under each graph, respectively, for the tested foetal brains and placentas per group. Statistics were done with Kruskal-Wallis and Dunn's multiple comparison post hoc test; ^∗^*p* < 0.05.

As previous results showed an increase in gene expression of hypoxia-inducible factors (HIFs) upon hsFLT1 overexpression in PE placentas [12], pimonidazole was injected into pregnant mice to visualize hypoxia at 18.5 dpc. Immunohistochemical staining with antipimonidazole was performed to elucidate the effects of hsFLT1 on the oxygen supply of the placenta (Figures 2(a) and 2(b)). Quantification of pimonidazole-positive area (equivalent to the amount of hypoxia in the tissues) indicated a tendency of increased hypoxia in the PE wt group (maternal hsFLT1 expression) in total placental area as well as in the different placental compartments, spongiotrophoblast, and labyrinth as well as in the decidua, when compared to the control group (Ctrl). Notably, the decidua and spongiotrophoblast showed a strong staining intensity compared to the labyrinth (Figure 2(b)). But here, it is mentioned that the labyrinth compartment is mainly composed of maternal and foetal vessels facing each other, and cell density itself is lower than in the spongiotrophoblast or decidua. Also, increased hypoxia was observed upon systemic hsFLT1 investigating foetal brain tissues compared to Ctrl tissues by pimonidazole staining (data not shown).

To investigate the effect of hsFLT1 on the expression of oxidative-stress/hypoxia-responsive markers, *Hemeoxygenase-1* (*HO-1*) expression was examined at 18.5 dpc in placental and foetal brain tissue. Transcript expression of *HO-1* was significantly upregulated in placental tissues of PE wt and PE het compared to the Ctrl group (Figure 2(c)). No significant difference in *HO-1* mRNA expression could be found between Ctrl and PE groups in foetal brain tissues (Figure 2(f)). In contrast, HO-1 protein expression was significantly increased in foetal brain tissues of PE het foetuses and increased by trend in PE wt foetal brains compared to both control groups (Ctrl and Dox Ctrl) (Figures 2(g) and 2(h)). Also, in placental tissues, HO-1 protein levels tended to be increased for both PE groups compared to controls (Figures 2(d) and 2(e)). In conclusion, systemic hsFLT1 overexpression led to an upregulation of hypoxic areas in placentas with elevated levels of the hypoxia marker HO-1 especially in foetal brains but also in placentas with maternal and fetoplacental hsFLT1 impact.

### 3.3. Preeclampsia-Like Symptoms during Pregnancy Result in a Reduction of Brain Weight and Volume in Asymmetrical Growth-Restricted Foetuses

**Figure 3:**
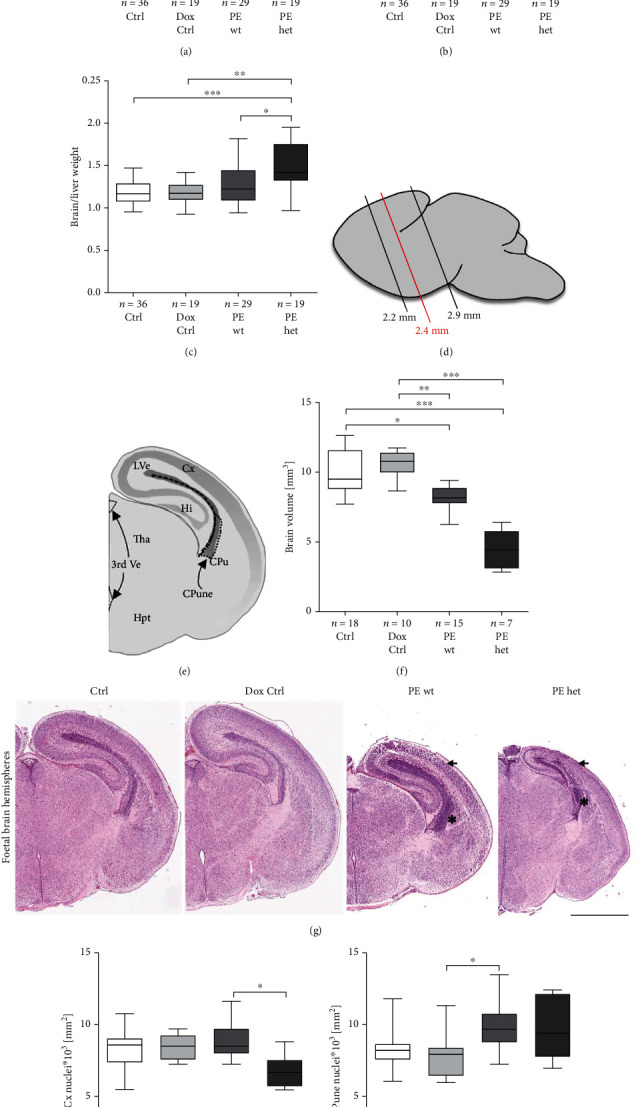
Morphological foetal brain characteristics upon systemic human sFLT1 (soluble fms-like tyrosine kinase-1) overexpression at the end of the pregnancy. (a) At the end of the pregnancy, the brain weight was reduced upon exclusive maternal (PE wt) as well as maternal and fetoplacental (PE het) hsFLT1 overexpression compared to both controls (Ctrl and Dox Ctrl). (b) In Ctrl and Dox Ctrl foetuses, the brain weight was proportional to the body weight, which was mostly true also for PE wt foetuses. In contrast, in PE het foetuses, the brain weight was partially preserved from the weight reduction and thus, the brain to body weight ratio was increased. (c) The same tendency was seen in the brain to liver weight ratio, which was increased in PE het foetuses and tended to be increased in PE wt foetuses compared to both controls. (d) Schematic overview of the region used for brain volume measurement in black and region of representative images highlighted in red. Brain areas (mm^2^) were measured in duplicate every 100 *μ*m between +2.20 mm and +2.90 mm rostral, and the volume (mm^3^) was quantified for the whole region including the different brain regions. (e) Cx: cortex; Hi: hippocampus; Tha: thalamus; Hpt: hypothalamus; CPu: caudate putamen; CPune: caudate putamen neuroepithelium; LVe: lateral ventricle; 3rd Ve: third ventricle. (f) The brain volume was reduced upon both types of hsFLT1 overexpression (PE wt and PE het) compared to both controls. (g) Representative haematoxylin-eosin-stained coronal sections in the region ~+2.40 mm rostral. Scale bar: 2 mm. The cellular density (nuclei∗10^3^ per mm^2^) was quantified in the cortex and the caudate putamen neuroepithelium. (h) Reduced cellular density was seen in the cortex (arrow) in PE het foetuses, whereas it was slightly increased in PE wt foetuses compared to both controls (Ctrl and Dox Ctrl). (i) In contrast, in the caudate putamen neuroepithelium (asterisk), the cellular density was increased in PE wt and PE het foetuses compared to foetuses of the Ctrl and Dox Ctrl group. Data are presented as box plot with median and interquartile range ± upper/lower extreme; sample size *n* of individual tested foetuses is listed under each graph, respectively; Kruskal-Wallis combined with Dunn multiple comparisons test; ^∗^*p* < 0.05, ^∗∗^*p* < 0.01, and ^∗∗∗^*p* < 0.001.

To elucidate the effect of hsFLT1 upregulation on foetal brain development, the brain weight and general brain morphology were assessed at the end of the pregnancy. At 18.5 dpc, the brain weight was reduced upon both types of hsFLT1 overexpression in PE wt and PE het foetuses compared to controls (Ctrl and Dox Ctrl) (Figure 3(a)). In PE wt foetuses, the reduction of the brain weight was mostly proportional to the body weight, whereas in PE het foetuses, the brain to body weight ratio was increased (Figure 3(b)). Nevertheless, the brain to liver weight ratio was increased in PE het foetuses and tended to be increased in PE wt foetuses, which indicated asymmetrical growth restriction in both types of hsFLT1 overexpression in the PE group (Figure 3(c)). A morphometrical analysis of different brain compartment areas was performed (Figure 3(d)). The analysed regions are displayed in representative haematoxylin-eosin-stained coronal sections ~+2.40 mm rostral (Figure 3(g)), including the cortex (Cx), the hippocampus (Hi), the thalamus (Tha), the hypothalamus (Hpt), the caudate putamen (CPu), the caudate putamen neuroepithelium (CPune), the lateral ventricles (LVe), and the third ventricle (3rd Ve) (Figure 3(e)). The brain volume was reduced in both types of systemic hsFLT1 overexpression (PE wt and PE het) (Figure 3(f)). Furthermore, a reduced number of nuclei per square millimeter in the cortex of foetal brains in the PE het group (Figure 3(h)) and an increased number of nuclei per square millimeter in the caudate putamen neuroepithelium in PE wt and PE het brains were detected (Figure 3(i)).

To conclude, foetuses of hsFLT1-induced experimental PE pregnancies showed tendencies of an asymmetrical FGR in both types of hsFLT1 overexpression with increasing preservation of the brain mass with advancing FGR.

### 3.4. Growth-Restricted Foetuses of Preeclamptic Pregnancies Exhibited Reduced Cortical Area and Increased Neuroepithelial Area in the Caudate Putamen Region

**Figure 4:**
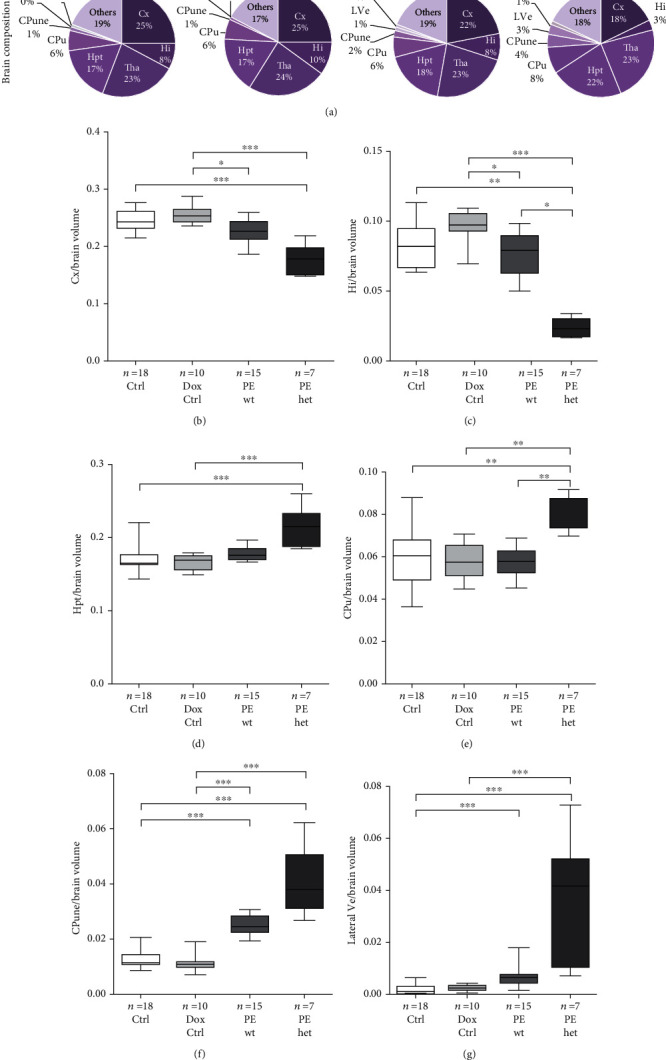
Detailed volumetric analyses of foetal brain regions. (a) Percentual composition of the different brain regions. Cx: cortex; Hi: hippocampus; Tha: thalamus; Hpt: hypothalamus; CPu: caudate putamen; CPune: caudate putamen neuroepithelium; LVe: lateral ventricles; 3rd Ve: third ventricle; others: include habenular nuclei, fimbria hippocampus, internal capsule, optic tract, and amygdala. (b) The cortical area as well as (c) the hippocampal area normalized to the total brain area was decreased upon combined maternal and fetoplacental human soluble fms-like tyrosine kinase-1 (hsFLT1) overexpression (PE het) and tended to be decreased upon maternal hsFLT1 overexpression (PE wt) compared to both controls (Ctrl and Dox Ctrl). Normalized to the total brain area, the area of (d) the hypothalamus and (e) the caudate putamen was increased upon combined maternal and fetoplacental hsFLT1 overexpression (PE het) but not upon maternal hsFLT1 overexpression (PE wt). Furthermore, (f) the neuroepithelium of the caudate putamen region, as well as (g) the lateral ventricles showed an increased volume in both types of hsFLT1 overexpression (PE wt and PE het). Data are presented as pie chart displaying the mean percentage of each brain region or box plot with median and interquartile range ± upper/lower extreme; sample size *n* of individual tested foetal brains is listed under each graph, respectively; Kruskal-Wallis combined with Dunn multiple comparisons test; ^∗^*p* < 0.05, ^∗∗^*p* < 0.01, and ^∗∗∗^*p* < 0.001.

To reveal differing influences on specific brain regions, the volume of each brain region was analysed in more detail. Overall, upon both types of hsFLT1 overexpression during pregnancy (PE wt: exclusive maternal and PE het: maternal and fetoplacental), differences in the percentual compositions of the foetal brains were observed compared to both controls (Ctrl and Dox Ctrl) (Figure 4(a)). The cortical and hippocampal area was decreased upon systemic hsFLT1 expression in PE wt and PE het foetuses (Figures 4(b) and 4(c)).. In contrast, the area of the hypothalamus, the caudate putamen, and the neuroepithelium of the caudate putamen region, as well as the lateral ventricles, were increased upon systemic hsFLT1 overexpression (PE wt and PE het) compared to controls (Ctrl and Dox Ctrl) (Figures 4(d)–4(g)).

In conclusion, brains of growth-restricted foetuses exhibited conservation of some brain regions, whereas the cortex and hippocampus were underrepresented and notably, the neuroepithelium and the ventricles were surpassingly large. This phenotype indicates adverse influences of the hsFLT1 overexpression on cell migration and cortical expansion.

### 3.5. Iron Deposits Are Increased in Foetal Brains of Preeclamptic Pregnancies

**Figure 5:**
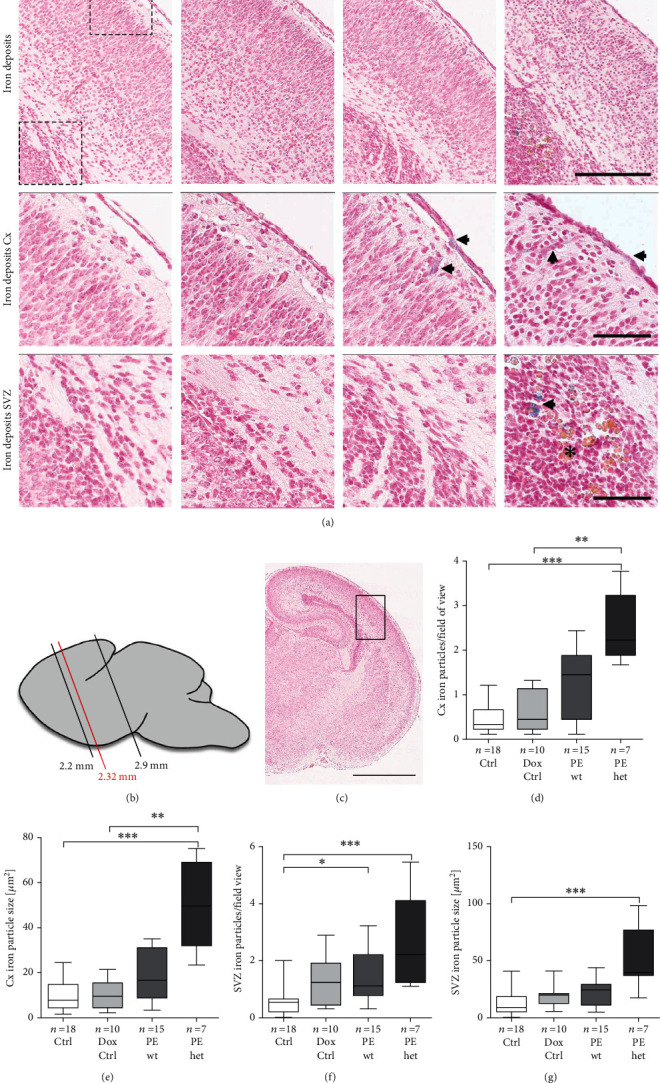
Iron presence in the pia mater and caudate putamen of foetal brains upon systemic human sFLT1 (soluble fms-like tyrosine kinase-1) overexpression. (a) Representative images of Perls Prussian Blue- (Perls) stained coronal brain sections with a general overview containing the cortex (Cx) and subventricular zone (SVZ) (upper panel; scale bar: 200 *μ*m), as well as detailed views of the Cx and SVZ (middle and lower panel; scale bar: 60 *μ*m). In the Cx, iron deposits (arrow) were present in small vessels, which rise from the pia mater. In the SVZ, iron deposits (arrow) were present in regions with high erythrocyte content (asterisk). (b) Schematic representation of the analysed region (~2.32 mm rostral, red) for Perls. (c) Overview of Perls reaction on foetal brain with the highlighted region of detail view of (a) first row. Scale bar: 1 mm. Iron deposits per field of view within (d) the Cx and (f) the SVZ, as well as iron particle size in (e) the Cx and (g) SVZ, were increased especially in PE het foetuses compared to controls (Ctrl/Dox Ctrl). Data are presented as box plot with median and interquartile range ± upper/lower extreme; sample size *n* of individual tested foetal brains is listed under each graph, respectively; Kruskal-Wallis combined with Dunn multiple comparisons test; ^∗^*p* < 0.05, ^∗∗^*p* < 0.01, and ^∗∗∗^*p* < 0.001.

Since previous results by us indicated disrupted blood flow into and via the placental barrier upon hsFLT1 overexpression in PE mice [11, 12], an influence on the brain vasculature was assumed. As a first hint of possible vascular defects, the Perls Prussian Blue (Perls) reaction was performed to detect free iron deposits as a sign for haemorrhages or microbleedings in the foetal brain (Figure 5(a)). The analysed brain region (Figure 5(b)) and the location of the selected representative images (Figure 5(c)) are highlighted. Iron deposits were only present in the proximity of vessels and regions with high erythrocyte presence, for example, the pia mater in the proximity of the cortex (Cx) and the subventricular zone (SVZ) region (Figure 5(a)). The number of iron deposits per field of view (Figures 5(d) - 5(f)) as well as the iron particle size (Figures 5(e) - 5(g)) within the cortex and the SVZ was increased especially in PE het foetuses compared to both controls (Ctrl and Dox Ctrl).

Hence, the presence of free iron in the pia mater and cortex as well as haemorrhages in the caudate putamen could be signs of impaired microvascularization, endothelial dysfunction of existing blood vessels, or disruption of newly generated and thus highly vulnerable blood vessels due to blood pressure changes.

### 3.6. Changed Expression Profile of Brain-Specific and Angiogenesis Markers in Foetal Brain Tissues of Preeclamptic Pregnancies Indicates Brain Injury

**Figure 6:**
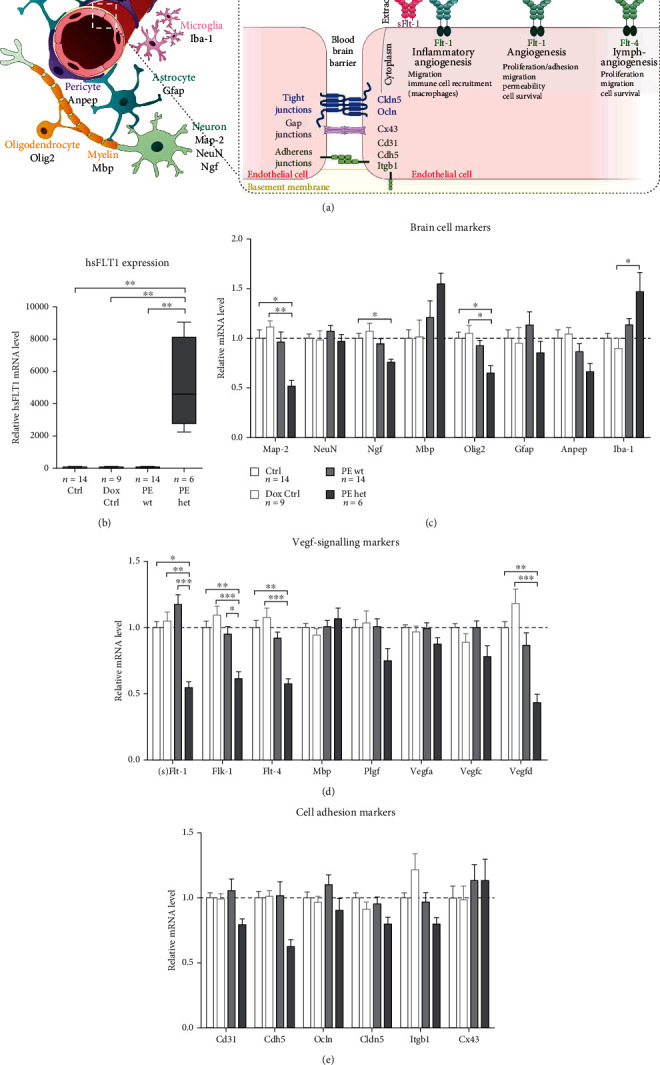
Marker gene expression in foetal brain tissue upon systemic human sFLT1 (soluble fms-like tyrosine kinase-1) overexpression. (a) Schematic illustration of the location and interaction of the analysed markers for specific brain cell types and marker genes of the Vegf-signalling pathway as well as the blood-brain barrier (BBB). (b) *hsFLT1* mRNA expression analysis could prove exclusive hsFLT1 expression in foetal PE het brains, with no expression in PE wt brains or controls (Ctrl and Dox Ctrl). (c) mRNA level of marker genes for different brain cell types like *Microtubule Associated Protein 2* (*Map-2*), *Neuronal Nuclei* (*NeuN*), *Nerve Growth Factor* (*Ngf*), *Myelin Basic Protein* (*Mbp*), *Oligodendrocyte Transcription Factor 2* (*Olig2*), *Glial Fibrillary Acidic Protein* (*Gfap*), *Alanyl Aminopeptidase* (*Anpep*), and *Ionized Calcium-binding Adapter Molecule 1* (*Iba-1*). (d) mRNA levels of members of the Vegf-signalling pathway *soluble Fms-related tyrosine kinase 1* (*sFlt-1*) *foetal liver kinase 1* (*Flk-1*), *placental growth factor* (*Plgf*), and *vascular endothelial growth factor* (*Vegfa*, *Vegfb*) as angiogenesis and *Fms-related tyrosine kinase 4* (*Flt-4*), *Vegfc*, and *Vegfd* as lymph-angiogenesis markers. (e) mRNA levels of members of different adhesion molecules, which are involved in blood-brain barrier maintenance like the *Cluster of Differentiation* 31 (*Cd31*), *Cadherin 5* (*Cdh5*), *Occludin* (*Ocln*), *Claudin 5* (*Cldn5*), *Integrin-β1* (*Itgb1*), and *Connexin 43* (*Cx43*). Data are presented as mean ± standard error of the mean normalized to *glyceraldehyde-3-phosphate dehydrogenase* (*Gapdh*) and *β-Actin* (*Actb*) as housekeeping genes and final gene expression analysis done by the ∆∆CT method. Sample size *n* is listed under each graph, respectively, for the tested foetal brains per group. Statistics was done with Kruskal-Wallis and Dunn's multiple comparison post hoc test; ^∗^*p* < 0.05, ^∗∗^*p* < 0.01, and ^∗∗∗^*p* < 0.001.

To investigate the cellular composition and influences on the brain vasculature, including the blood-brain barrier (BBB), specific cell markers for different neural cells (neurons and glial cells) as well as marker genes for (lymph-)angiogenesis and adhesion molecules at the BBB, were analysed on mRNA level (Figure 6(a)). Of note, only the PE het group showed *hsFLT1* mRNA expression, with no detectable *hsFLT1* mRNA in foetal brain tissues in the Ctrl, Dox Ctrl, and PE wt group (Figure 6(b)), and no hsFLT1 protein was detectable in serum samples of PE wt foetuses (data not shown). Upon maternal and fetoplacental hsFLT1 overexpression (PE het), mRNA levels of the neuronal markers *Microtubule Associated Protein 2* (*Map-2*) and *Nerve Growth Factor* (*Ngf*) and the oligodendrocyte marker *Oligodendrocyte Transcription Factor 2* (*Olig2*) were significantly lower and of *Glial Fibrillary Acidic Protein* (*Gfap*) as a marker for astrocytes and *Alanyl Aminopeptidase* (*Anpep*), as a pericyte marker, tended to be lower compared to controls (Ctrl and Dox Ctrl). In contrast, mRNA levels of *Myelin Basic Protein* (*Mbp*), as a marker for mature/myelinating oligodendrocytes and *Ionized Calcium-binding Adapter Molecule 1* (*Iba-1*), as a marker for microglia, were increased in foetal PE het brains in comparison to both controls (Ctrl and Dox Ctrl). Additionally, in foetal PE wt brains, mRNA levels of *Mbp*, *Gfap*, and *Iba-1* tended to be increased compared to controls, whereas the mRNA level of *Anpep* tended to be lower in PE wt compared to controls (Figure 6(c)). In the context of (lymph-)angiogenesis, mRNA levels of the members of the Vegf-signalling pathway like the endogenous receptors (*soluble*) *Fms-like tyrosine kinase-1* (*(s)Flt-1*), *foetal liver kinase-1* (*Flk-1*), *Flt-4*, and their corresponding binding partners *vascular endothelial growth factors* (*Vegfa-d*) as well as the *placental growth factor* (*Plgf*) were analysed. Regarding the endogenous murine variant of *(s)Flt-1*, differences in the regulation were observed depending on the origin of the human sFLT1 (hsFLT1) overexpression. In foetal PE wt brains *(s)Flt-1*, mRNA levels were slightly increased, whereas in foetal PE het brains, *(s)Flt-1* mRNA levels were significantly lower compared to both controls (Ctrl and Dox Ctrl). In contrast, the other two membranous receptors of the Vegf-family *Flk-1* and *Flt-4* were significantly lower in the PE het group and tended to be lower in the PE wt group, indicating similar regulation types in both hsFLT1 overexpression origins but in different severity stages. Concerning the corresponding binding partners, in the PE het group, *Vegfa-d* mRNA levels were lower compared to controls, with only *Vegfd* significantly lower expressed which could be also seen in the PE wt group (Figure 6(d)). Regarding adhesion molecules involved in the integrity of the BBB, the endothelial cell marker *Cluster of differentiation 31* (*Cd31*), the tight junction proteins *Claudin 5* (*Cldn5*) and *Occludin* (*Ocln*), the adherens junction proteins *Cadherin 5* (*Cdh5*) and *Integrin-β1* (*Itbg1*), as well as the gap junction protein *Connexin 43* (*Cx43*), were analysed. In foetal PE het brains, mRNA levels of *Cd31*, *Cdh5*, *Ocln*, *Cldn5*, and *Itgb1* tended to be reduced compared to both controls (Ctrl and Dox Ctrl), whereas foetal PE wt brains seemed to be unaffected (Figure 6(e)).

To sum up, especially in foetal brains with direct impact of the hsFLT1 overexpression (maternal and fetoplacental hsFLT1), adverse influences on the mRNA expression of angiogenic and lymphatic markers, as well as adhesion molecules at the BBB, were exhibited, combined with reduced neuronal marker gene expression but increased expression of markers induced by brain injury. Even if less pronounced, in some parts, this also holds true in foetal brains with indirect impact of the hsFLT1 overexpression (exclusive maternal hsFLT1 origin). This overall indicates cerebral endothelial dysfunction and brain damage due to direct and indirect influences of the hsFLT1 overexpression during pregnancy.

## 4. Discussion

The current study focused on the direct and indirect influences of increased antiangiogenesis by systemic (exclusive maternal or combined maternal and fetoplacental) human sFLT1 (hsFLT1) overexpression on foetal brain development in PE-affected murine pregnancies.

Here we confirmed the increased utero-placental under-perfusion upon systemic hsFLT1 overexpression by an increase in the hypoxia marker staining using pimonidazole as we already shown recently analysing HIF expression amongst others [11; 12]. Especially the spongiotrophoblast and the maternal decidua compartment seems to be mostly affected by reduced oxygenation upon systemic sFLT1 increase in PE wt placentas (maternal overexpression) confirming previous results [28]. Moreover, the reduced utero-placental oxygenation in hsFLT1-induced PE pregnancies resulted in increased mRNA and protein levels of HO-1 in placentas and foetal brains mostly upon combined maternal and feto- placental hsFLT1 overexpression. HO-1 as a stress-inducible enzyme exhibits protective functions against oxidative stimuli [29]. Cudmore et al. showed that HO-1 inhibited sFLT1 release in human PE in placental villous explants and in endothelial cells providing evidence for a protective function in pregnancy [30]. However, regarding the HO-1 expression levels in PE there still exist contrary results [29]. Studies in the reduced uterine perfusion pressure (RUPP) placental ischemia rat model revealed a significant increase in HO-1 production in placentas of RUPP animals compared with control animals, which is associated with increased levels of rat endogenous sFlt-1 [31]. This is in accordance with our results shown in this study. However, if HO-1 really acts here as a protective molecule to attenuate the adverse effects upon sFLT1 expression has to be addressed in further studies.

Regarding the effect of systemic hsFLT1 overexpression on foetuses, we revealed different severity levels of FGR, leading to viable and nonviable foetuses depending on the type of hsFLT1 overexpression. Thereby, the direct influence of hsFLT1 overexpression (combined maternal and fetoplacental) led to more pronounced adverse effects than the indirect influence due to exclusive maternal hsFLT1 overexpression. Upon both types of systemic hsFLT1 overexpression, foetuses revealed an asymmetrical FGR displayed by an increased brain/liver weight ratio. An asymmetrical FGR is mostly caused by extrinsic influences like placental insufficiency combined with undernutrition occurring in later gestation [32] and is distinguished by reduction of the abdomen (mainly the liver), whereas the head and therefore the brain are relatively retained, a phenotype which is the case in our study. This phenomenon is often associated with the brain-sparing effect, in which the foetus adapts its circulation to maintain oxygen and nutrient supply to the brain [33], detectable via ultrasound Doppler sonography. The brain to liver ratio is thought to be relatively independent of the gestational time point and provides a stable measurement of FGR-type independent of foetal body weight [34–36]. According to human studies, about 80% asymmetrical growth-restricted infants catch up growth within the first year [37, 38], thought to be a compensatory mechanism following FGR [39]. Nevertheless, the brain-sparing effect is still a matter of debate regarding its protectiveness. Initially, it has been defined as a protective adaption mechanism of the brain to preserve blood circulation but an increased amount of studies describe an association of brain-sparing occurring during pregnancy with poor cognitive function or lower intelligence quotients in the offspring [40]. Thus, the increased brain/liver weight ratio shown in PE and FGR-affected foetuses in this study could be a sign of brain-sparing; however, this has to be analysed in detail in future experiments by ultrasound Doppler imaging of the cerebral arteries. In humans, preterm infants are at high risk to develop cognitive deficits or other neurological complications in later life. This association was also observed in preterm infants of PE pregnancies, with FGR seen as an additional risk factor [1, 16, 41–46]. In a study of Córcoles-Parada et al., with 196 children born very preterm combined with very low birth weight, children revealed a lower intelligence quotient, decreased grey and white matter integrity, and reduced cortical thickness at the age of 8-16 years [45]. In another study by Tokariev et al., with children of about 7 years old and born before 28 weeks of gestation, showed weaker memory-related brain activations in functional magnet resonance imaging and altered white matter microstructure in diffusion tensor imaging even with normally appearing cognitive abilities [44, 46].

All these findings pointed to a massive effect of preterm birth and FGR on brain development. In our current study, upon systemic hsFLT1 overexpression, the foetal brain weight and volume were reduced compared to controls. The highest impact was seen in the reduction of the cortical and hippocampal region and overrepresentation of the neuroepithelial region especially in the subventricular zone of the caudate putamen, which was further associated with a reduced cell density in the cortex and an increased cell density in the caudate putamen. These results could be verified by Liu et al. in a preeclampsia-FGR-like rat model using L-NAME (N*ω*-nitro-L-arginine methyl ester), a nitric oxide synthase (NOS) inhibitor [47]. At postnatal day 0 (P0), body and brain weight as well as cortical thickness was reduced in the L-NAME group compared to the control group, which strengthens the finding of delayed brain development upon PE. Interestingly, the authors also observed a decrease in progenitor cell proliferation in the neocortex upon L-NAME administration during pregnancy. In the offspring (P56), reduced neurogenesis in the cortex and hippocampus could be shown. Regarding the results found in the caudate putamen, a clinical study revealed an increased volume in the caudate putamen region in adults with autism spectrum disorder (ASD) [48]. Moreover, it is known that preeclampsia is associated with an increased risk for ASD in the offspring [49]. During foetal brain development, the ganglionic eminence (in later developmental stages, the caudate putamen neuroepithelium) is a transitory structure that guides cell and axon migration [50]. Neuroepithelial cells are the stem cells of the central nervous system, which generate the intermediate progenitor cells, known as radial glial cells, that differentiate into neurons and glia cells in the process of neurogenesis [50]. Consequently, progenitor cells proliferate in the caudate putamen neuroepithelium region and migrate to the cortex or hippocampal region, where they differentiate to neurons and glia cells. The process of cell migration in mice starts around the day 11 in pregnancy (11.0 dpc) [50]. This vulnerable time point of cell migration starts shortly after the induction of systemic hsFLT1 overexpression simulated in the hsFLT1/rtTA mouse model in this study (10.5 dpc). Furthermore, during foetal brain development at earlier time points (10.0-14.5 dpc), the ventricles occupy a large space, which becomes smaller with increasing cell proliferation and migration into the cortex [51]. In 2014, Carver et al. analysed the postnatal brain development in 6-month-old PE-affected offspring of pregnant CD-1 mice, which were injected with a sFlt-1 adenovirus starting at 8.0 dpc and the influences of pravastatin therapy. Here, it could be shown that PE/sFlt-1-affected offspring revealed reduced cell counts in the neocortex but combined with an increase of the total neocortical area, which was both ameliorated due to pravastatin treatment during pregnancy [52]. Even if the analysed time point is not the same, the reduced cell number in the cortex was similar to the results observed in our study. To conclude, the findings in our study reveal first insights into impaired and delayed cortical and hippocampal formation upon systemic hsFLT1 overexpression in mice. This could be due to reduced migration capacity of cells which proliferated in the neuroepithelium and thus reduced cortical expansion combined with partly enlarged ventricles. Moreover, since the development of the cerebral cortex is unique to mammals and is highly conserved throughout species, observations made in mouse models are applicable to humans, even if the time points are not the same between mouse and human [53]. Up to now, such a migration impairment has not been found in severe FGR infants or FGR stillbirths in human. In a pathological study of 63 third trimester stillbirth cases, the most common form of brain injury was grey matter damage with neuronal necrosis not investigating migration capacities of progenitor cells [54]. Moreover, a study by Kono et al. published interesting results for FGR from early-onset hypertension disorders of pregnancy (HDP) depending on their intrauterine growth status and gestational age at birth [55]. These results suggest that earlier onset of HDP with SGA (short for gestational age) may affect the death, cerebral palsy, and developmental delay at 3 years of age. Therefore, the results revealed in the current study might also yield to new insights into cortical expansion capacity in foetal brains of PE- and sFLT1-affected offspring observed in the human PE infants.

Gene expression analyses in foetal brains regarding neuronal and glial marker genes, marker genes involved in the angiogenesis Vegf-signalling pathway, or specific cell adhesion markers at the blood-brain barrier (BBB) were performed to reveal changes in the cellular composition and function at the neurovascular unit. For most analysed markers, both types of systemic hsFLT1 overexpression (maternal or maternal/fetoplacental) exhibited the same tendency but with different effect strengths. Therefore, the observed influences on mRNA expression profiles, even though not verified on protein level so far, seem to be mostly influenced by indirect factors, like placental dysfunction and impaired oxygen/nutrient supply to the developing brain compared to antiangiogenic effects directly affecting the foetal brain. In this study, we could show that *Olig2* mRNA levels, as a marker for oligodendrocytes (the myelin-forming cells) and their precursor cells, were reduced upon elevated hsFLT1 levels which could be related to an impaired proliferation and differentiation of the oligodendrocytes and their precursors. In mice, oligodendrogenesis starts around 12.5/13.5 dpc in the ventricular zone of the embryonic neural tube (ganglionic eminence). By 15.5 dpc, oligodendrocyte precursor cells began to disperse away from the ventricle into developing grey and white matter and after ~17.5 dpc, Olig2-positive cells are distributed across all different brain regions, where they differentiate into myelinating oligodendrocytes, which start to express the myelin basic protein (Mbp) [56, 57]. A recent study by Davidson et al. could show that upon ischemic conditions during the last part of the pregnancy in sheep, at term, the foetal brains exhibited less Olig2-positive cells [58]. Interestingly, we also revealed beside a decreased *Olig2* expression an elevated level of *Mbp* a marker for oligodendrocyte myelin production, which contrasted with the study in foetal sheep, which found reduced Mbp levels [58]. In mice and humans, myelination starts shortly before birth with its highest activity in the postnatal brain development [53]. Thus, the increased *Mbp* mRNA levels combined with the reduced *Olig2* mRNA levels upon hsFLT1 overexpression in the current study could be a sign of reduced oligodendrocytes and their progenitor cells, but which might have a higher myelin-forming activity, a phenomenon which was recently described *in vitro* under reduced oligodendrocyte cell densities [59]. Nevertheless, unlike most progenitor cells, oligodendrocyte progenitor cells remain also in the adult brain, where they retain the ability to generate new oligodendrocytes, that allow myelin regeneration also in adult stages [57]. Hence, we speculate that reduced numbers of oligodendrocytes and their progenitor cell at the end of the pregnancy may lead to an adverse myelin regeneration capacity, resulting in a negative impact on the white matter substance and possible long-term complications.

Regarding the microglia development, overexpression of hsFLT1 induced an upregulation of *Iba-1* mRNA, as a marker for brain resident immune cells, in 18.5 dpc foetal brains. The developmental origin of microglia is still not finally proven. Nevertheless, it is assumed that they originate from macrophages of the yolk sac, which colonize the brain during prenatal development, starting around 10.0 dpc [60]. However, an upregulation of *Iba-1* mRNA does not necessarily mean that it leads to an inflammatory response. Microglia are highly dynamic cells, and their activation can be functionally differentiated to a proinflammatory M1-phenotype or an alternative anti-inflammatory M2-phenotype. M2-microglia typically secrete anti-inflammatory cytokines and growth factors which rather promote repair and regeneration of damaged tissue, whereas M1-microglia specifically release proinflammatory cytokines and chemokines and contribute to neurodegeneration [61]. To check which phenotype is predominant in offspring of our PE model, cellular distribution and morphology of microglia as well as the expression of pro- and anti-inflammatory cytokine chemokines and growth factors need to be characterized in future studies. Interestingly, the mRNA expression of *Gfap*, as a classical marker for astrocytes, was exclusively downregulated in foetal brains upon combined maternal and fetoplacental hsFLT1 overexpression, whereas it tended to be increased upon exclusive maternal hsFLT1 overexpression. Astrocytes have multiple functions not only in the developing brain by modulation of energy metabolism and inflammation but also for the integrity of the neurovascular unit [62, 63]. The neurovascular unit is a complex interacting system of diverse cell types, including neurons, astrocytes, and microglia, as well as vascular cells including pericytes, vascular smooth muscle cells, and endothelial cells combined with the BBB [64]. Pericytes are multifunctional cells embedded within the vascular wall and can be detected by *Alanyl Aminopeptidase* (*Anpep*) [65]. Even if not significant, on mRNA level, *Anpep* was reduced upon hsFLT1 overexpression, which could be a hint for reduced pericyte presence at the neurovascular unit. Moreover, there are hints that both astrocytes and pericytes are important for the preservation of tight junctions at the BBB by regulation of proteins like Occludin or Claudin 5, as well as regulation of the cerebral blood flow and brain angiogenesis [62, 64]. As we observed only a tendency of downregulation exclusively upon combined hsFLT1 overexpression (maternal/fetoplacental) of different markers for BBB on mRNA level such as *Cluster of Differentiation* 31 (*Cd31*), *Cadherin 5* (*Cdh5*), *Occludin* (*Ocln*), *Claudin 5* (*Cldn5*), and *Integrin-β1* (*Itgb1*), future studies are needed to validate the relationship between downregulation of astrocyte and pericyte markers and markers of BBB in the foetal and postnatal brain at different developmental timepoints. Also, Zhang et al. revealed disturbed integrity of the BBB due to loss of tight junction proteins in neonatal and adult mouse models of cerebral ischemia [66]. In a PE rat model, it is shown that PE induced a cascade of neuroinflammation and oxidative injury to the BBB [67], which is in accordance with the findings in this study. As shown in Lara et al.'s study, cognitive alterations present in children born from preeclamptic pregnancies are strongly associated with impaired cerebral Vegf levels and changed angiogenesis phenotype, a finding which is also seen in this study with lower levels of *Vegfd* upon hsFLT1 overexpression [25]. This is in contrast to a rat model with cortical cold injury, in which an upregulation of Vegfd was associated with BBB breakdown and angiogenesis [68]. Here in this study, we detected free iron deposits in foetal brain upon hsFLT1 overexpression, which goes in line with the increased expression of HO-1 by elevated catabolism of heme and could be signs of microbleedings. Hence, it seems plausible to speculate that sFLT1-overexpression is associated with a disturbed angiogenesis or increased fragility of newly formed vessels, which needs to be proven in future studies.

Overall, this study revealed first hints that hsFLT1 overexpression in PE-affected murine pregnancies impaired not only placental perfusion and function but also foetal brain development by changing the neurovascular integrity and the cerebral angiogenic state. Whether in our study the PE- and hsFLT1-affected foetuses (direct or indirectly) also show less vascularization in the brain is still not finally proven and needs further investigations.

Taken together, regarding most analysed features, our results exhibited same tendencies but with increasing severity level of adverse brain development upon both types of hsFLT1 overexpression: (1) indirect: exclusive maternal hsFLT1 overexpression by influencing placental perfusion and nutrient/oxygen supply to the developing foetus and foetal brain and (2) direct: combined maternal and fetoplacental hsFLT1 overexpression by additionally systemically affecting the foetal angiogenic capacity.

## 5. Conclusions

Evaluation of adverse effects of human sFLT1 (hsFLT1) overexpression on foetal brain development indicated a delayed cell migration from the caudate putamen neuroepithelium to the cortex and thus reduced cortical expansion in foetal brains upon hsFLT1 overexpression in preeclamptic pregnancies that suffered from impaired placental perfusion and reduced oxygenation. Thereby, this reduced cortical expansion was more distinct in foetuses, which were directly affected by the systemic hsFLT1 overexpression (maternal and fetoplacental hsFLT1 overexpression) and thus nonviable. Although less pronounced, cortical expansion was impaired likewise in viable foetuses, which were indirectly affected by exclusive maternal hsFLT1 overexpression. Furthermore, adverse influences on the formation and/or function of the brain vasculature were shown by the presence of free iron deposits and alterations in the expression profile of molecules involved in the Vegf-signalling pathway, which could be signs of cerebral microbleedings. Thereby, in viable foetuses, the iron deposits were mainly present in the pia mater, which could be due to adaptation of the foetal circulation to preserve oxygen and nutrient supply to the brain. All these results further let assume a negative influence of even exclusive maternal hsFLT1 overexpression during pregnancy also on the offspring's long-term neurodevelopment.

## Figures and Tables

**Table 1:** Maternal and foetal experimental groups.

Maternal groups		Foetal groups	
*Ctrl* Genotype: hsFLT1/rtTA	No hsFLT1 expression	*Ctrl* Genotype: hsFLT1/rtTA	No hsFLT1 expression

*Dox Ctrl* Genotype: hsFLT1	No hsFLT1 expression	*Dox Ctrl* Genotype: hsFLT1	No hsFLT1 expression

*PE* Genotype: hsFLT1/rtTA	Systemic hsFLT1 overexpression	*PE wt* Genotype: hsFLT1	Exclusive maternal systemic hsFLT1 overexpression
		*PE het* Genotype: hsFLT1/rtTA	Combined maternal and foetal systemic hsFLT1 overexpression

**Table 2:** Oligonucleotides used for gene expression analysis, genotyping, and sex determination.

Gene	NCBI number	Primer sequence (5′→3′)	Base pairs
Housekeeping genes			
*β-Actin*	NM_007393.5	For: CCTCTATGCCAACACAGTGC	206
		Rev: CCTGCTTGCTGATCCACATC	
*Gapdh*	XM_011241214.1	For: ACAACTCACTCAAGATTGTCAGCA	121
		Rev: ATGGCATGGACTGTGGTCAT	

Angiogenesis			
*hsFLT1*	XM_017020485.1	For: AATCATTCCGAAGCAAGGTG	221
		Rev: TTTCTTCCCACAGTCCCAAC	
*(s)Flt-1*	NM_001363135.1	For: TATAAGGCAGCGGATTGACC	159
		Rev: TCATACACATGCACGGAGGT	
*Flk-1*	NM_001363216.1	For: GGCGGTGGTGACAGTATCTT	162
		Rev: GTCACTGACAGAGGCGATGA	
*Flt-4*	NM_008029.3	For: GTGGCTGTGAAGATGCTGAA	199
		Rev: TGACACGCAAGAAGTTGGAG	
*Plgf*	XM_011244016.1	For: CGTCCTGTGTCCTTCTGAGT	200
		Rev: CCTCTTCCTCTTCCCCTTGG	
*Vegfa*	NM_001025257.3	For: CAGGCTGCTGTAACGATGAA	140
		Rev: GCATTCACATCTGCTGTGCT	
*Vegfb*	NM_011697.3	For: AACACAGCCAATGTGAATGC	157
		Rev: GGAGTGGGATGGATGATGTC	
*Vegfc*	NM_009506.2	For: CAAGGCTTTTGAAGGCAAAG	159
		Rev: TCCCCTGTCCTGGTATTGAG	
*Vegfd*	NM_001308489.1	For: CAACAGATCCGAGCAGCTTC	155
		Rev: AAAGTTGCCGCAAATCTGGT	

Hypoxia marker			
HO-1	NM_010442.2	For: CACGCATATACCCGCTACCT	175
		Rev: CCAGAGTGTTCATTCGAGCA	

Cell adhesion markers			
*Cd31*	NM_008816.3	For: ATGACCCAGCAACATTCACA	200
		Rev: CACAGAGCACCGAAGTACCA	
*Cdh5*	NM_009868.4	For: TCACCATTGAGACAGACCCC	233
		Rev: TGGCAGCTTGAAGTGGTAGA	
*Ocln*	NM_001360538.1	For: ACAGTCCAATGGCCTACTCC	162
		Rev: TACCATTGCTGCTGTACCGA	
*Cldn5*	NM_013805.4	For: CTTTGTTACCTTGACCGGCG	198
		Rev: CCCAGCTCGTACTTCTGTGA	
*Itgb1*	NM_010578.2	For: GAGACATGTCAGACCTGCCT	194
		Rev: TCCTTCTCCTTGCAATGGGT	
*Gja1 (Cx43)*	NM_010288.3	For: GAGGGGGTGAAGGAGTTTTC	233
		Rev: TGCAATGAAGCTGAACATGAC	

Brain cell type markers			
*Map-2*	NM_001310634.1	For: CGACACAGAACAGAGGGAGT	191
		Rev: TCTCAAACGGCTGGTGGTAT	
*NeuN*	NM_001039167.1	For: ACCCAAGCCTCAGTTAGCAT	187
		Rev: GTGGAGGAGATGGGGTTTGA	
*Ngf*	NM_013609.3	For: CAGTGTCAGTGTGTGGGTTG	206
		Rev: TGTGAGTCGTGGTGCAGTAT	
*Mbp*	NM_001025251.2	For: TGTTTCCTCTCAGAGCCCAG	246
		Rev: AGCGACTCGATTCAGTGACA	
*Olig2*	NM_016967.2	For: CCCCAGAACCCGATGATCTT	206
		Rev: GGTGCTGGAGGAAGATGACT	
*Gfap*	NM_001131020.1	For: AAGGTTGAATCGCTGGAGGA	205
		Rev: ACCACTCCTCTGTCTCTTGC	
*Anpep*	NM_008486.3	For: CAGGGCCTGTACATCTTCCA	161
		Rev: TCAATGTTAGGTGCCGGAGT	
*Iba-1*	NM_001361501.1	For: ATGCTGGAGAAACTTGGGGT	191
		Rev: CCAGTTGGCCTCTTGTGTTC	

Genotyping			
*hsFLT1*	NM_001159920.2	For: CAAGGACGTAACTGAAGAGG	465
		Rev: TTTCTTCCCACAGTCCCAAC	
*Col1a1*		For: CCATCCCAACAATACATCACA	200
		Rev: TGGTTTCTTTGGGCTAGAGG	
rtTA		For: AAAGTCGCTCTGAGTTGTTAT	
		Rev-wt: GGAGCGGGAGAAATGGATATG	650
		Rev-mut: GCGAAGAGTTTGTCCTCAACC	340

Sex determination			
*IL-3*	NM_010556.4	For: GGGACTCCAAGCTTCAATCA	544
		Rev: TGGAGGAGGAAGAAAAGCAA	
*Sry*	NM_011564.1	For: TGGGACTGGTGACAATTGTC	402
		Rev: GAGTACAGGTGTGCAGCTCT	

## Data Availability

The authors declare that all supporting methods are available within the article. The underlying data that support the findings of this study are available from the corresponding author on reasonable request.
